# Facilitators and Barriers to Take up a COVID-19 Vaccine Booster Dose among Community-Dwelling Older Adults in Hong Kong: A Population-Based Random Telephone Survey

**DOI:** 10.3390/vaccines10060966

**Published:** 2022-06-17

**Authors:** Zixin Wang, Yuan Fang, Fuk-yuen Yu, Paul Shing-fong Chan, Siyu Chen, Fenghua Sun

**Affiliations:** 1 Jockey Club School of Public Health and Primary Care, The Chinese University of Hong Kong, Hong Kong, China; benfyyu@cuhk.edu.hk (F.-y.Y.); pchan@link.cuhk.edu.hk (P.S.-f.C.); chensiyu@link.cuhk.edu.hk (S.C.); 2 Department of Health and Physical Education, The Education University of Hong Kong, Hong Kong, China; lunajoef@gmail.com (Y.F.); fhsun@eduhk.hk (F.S.)

**Keywords:** COVID-19 vaccine booster dose, uptake, older adults, facilitators and barriers, random telephone survey, China

## Abstract

A COVID-19 vaccine booster dose is effective and safe for older adults. This study investigated facilitators and barriers to take up a COVID-19 vaccine booster dose among older adults in Hong Kong. Participants were Chinese-speaking community-dwelling adults aged ≥65 years. Telephone numbers were randomly selected from up-to-date telephone directories. A total of 395 participants completed the telephone interview. Logistic regression models were fitted. Among the participants, 31.6% received a COVID-19 vaccine booster dose. After adjustment for significant background characteristics, positive attitudes toward the booster dose, perceiving significant others would support them to receive the booster dose, and less uncertainty regarding the choice of the booster dose was associated with higher uptake of a COVID-19 vaccine booster dose. Concerns about poorer responses to the booster dose due to older age and the presence of chronic conditions were negatively associated with the dependent variable. In addition, the belief that governmental promotional materials could address their concern and were helpful for them to make decisions was associated with a higher COVID-19 vaccine booster dose uptake. Improving booster dose health promotion materials, modifying perceptions, involving significant others and reducing uncertainty are potentially useful strategies to improve COVID-19 vaccine booster dose uptake among older adults.

## 1. Introduction

Globally, increasing age is a leading risk factor of COVID-19 severe cases and mortality [1]. Hong Kong has just been through the fifth wave of the COVID-19 outbreak, mainly caused by Omicron BA.2. The cumulative number of COVID-19 associated deaths was 9148 on 16 May 2022, and 96% of them were individuals aged 60 years or above (*n* = 8787) [2]. The crude case fatality ratio ranged from 0.4% in people aged 60–69 years, 1.8% among those aged 70–79 years, to 10.4% in those 80 years or above [2]. The majority (72.5%) of the COVID-19 associated deaths among older adults in Hong Kong occurred among those who had never received any COVID-19 vaccines [2].

COVID-19 vaccination is effective and safe for preventing severe consequences and deaths caused by COVID-19 among older adults [3]. However, there are concerns about the waning protection against COVID-19 after receiving the two-dose primary vaccine series [4,5,6,7,8]. Compared with people aged 18–49 years, the decline in humoral responses within six months after the second dose of COVID-19 vaccination was more significant among people aged 65 years or above [8]. Existing evidence showed that receiving the third dose of the COVID-19 vaccine, also known as a booster dose, could significantly increase immunogenicity to wild type and several variants of concern among healthy adults [5,6,9,10]. Recent studies showed that receiving a COVID-19 booster dose after the primary series increased antibody titers and neutralizing response among older adults without safety concerns [11,12]. Real-world evidence suggested that a booster dose provided the largest gain in protection among adults aged ≥65 years [13,14]. Unvaccinated older adults had 11.3–22.3 times the risk of confirmed infection, 19.5 times the risk of severe illness, and 61.4 times the risk of COVID-19-associated death compared with fully vaccinated older adults who received a booster dose [13,14]. Local data showed that three doses of Comirnaty (Fosun-BioNTech, equivalent to Pfizer-BioNTech outside China) and CoronaVac (Sinovac, Beijing, China) could reduce the risk of COVID-19 severe/fatal diseases and mortality by more than 97% among people aged 60 years or above [15]. Therefore, the World Health Organization and other international health authorities recommend a booster dose to protect vulnerable groups (e.g., older adults) and mitigate the negative impacts of COVID-19 on healthcare and the economy [16,17,18]. Hong Kong started to offer a free third dose of the COVID-19 vaccine on 11 November 2021 [19]. People aged ≥3 years who received Sinovac as primary series and those aged ≥12 who received Comirnaty as primary series are recommended to receive a third dose of COVID-19 vaccine 90 days after the completion of the second dose [20]. People could choose to receive the same brand or another brand of vaccine as a booster dose [20]. During the study period, there were no governmental incentives for receiving a COVID-19 vaccine booster dose. Receipt of a booster dose was not a prerequisite to enter public spaces.

People may be hesitant to receive a COVID-19 vaccine booster dose. At least eleven studies investigated willingness to receive a COVID-19 booster dose [21,22,23,24,25,26,27,28,29,30,31]. The prevalence of behavioral intention to receive a COVID-19 booster dose among the general population was 62–67% in the United States, 67.4–71% in Poland, and 93.7% in China [23,24,25,26,27]. Fewer studies looked at the uptake or acceptance of a COVID-19 booster dose among older adults. One study reported that 18.7 million persons aged 65 years or above in the United States received a COVID-19 vaccine booster dose from August to December 2021, accounting for 44.1% of people in this age group [32]. Studies in Poland showed that people aged 50 years or above had a higher intention to receive a COVID-19 vaccine booster dose than their younger counterparts (84.3% vs. 68.1%) [25,26]. However, people aged ≥50 years had a lower willingness to receive a COVID-19 vaccine booster dose compared to those aged 18–50 years in China (81.7% vs. 91.2–94.6%) [27]. There was a dearth of studies investigating facilitators and barriers to receive a COVID-19 vaccine booster dose among older adults. Only one report in the United States showed that ethnicity was associated with the uptake of a COVID-19 vaccine booster dose among people aged ≥65 years [32].

Across countries, history of seasonal influenza vaccination, perceived higher risk and severe consequences of COVID-19, belief in vaccine efficacy, and trust in information obtained from social media and significant others were found to be facilitators to receive primary COVID-19 vaccine series among older adults [33,34,35,36,37,38,39,40,41,42,43,44,45]. Barriers to receive primary COVID-19 vaccine series in this age group included concerns about safety and side effects [33,34,35,36,37,38,39,40,41,42,43,44,45]. These general facilitators and barriers might also influence the decision on whether to receive the COVID-19 vaccine booster dose among older adults in Hong Kong. Concerns about weaker protection and more severe side-effects among people with older age, the presence of chronic conditions that would decrease the protection of the vaccine, and concerns that COVID-19 vaccination would affect the control of chronic conditions were barriers to completing the primary COVID-19 vaccine series specific to older adults in Hong Kong [45]. It is likely that older adults in Hong Kong would have similar concerns when taking up the COVID-19 vaccine booster dose. Since there are two vaccines available as a booster dose, older people may be uncertain about which booster would be most suitable for them [46,47]. Such uncertainty was a barrier to complete primary COVID-19 vaccine series among older adults in Hong Kong [45]. In addition, the Hong Kong government has been actively promoting a COVID-19 booster dose through mass media channels. It is unclear whether such promotion caters to the needs of older adults. A previous study showed that satisfaction with governmental vaccine promotion materials was associated with higher completion of primary vaccine series among older adults in Hong Kong [45]. It is possible that satisfaction with governmental COVID-19 vaccine booster dose promotional materials is also a facilitator of COVID-19 vaccine booster dose uptake among local older adults.

This study investigated facilitators and barriers to receive a COVID-19 vaccine booster dose among a random sample of community-dwelling persons aged 65 years or above in Hong Kong. We hypothesized that older people who had supportive perceptions of the booster dose, with less uncertainty when choosing a booster dose, and a higher level of satisfaction with governmental booster dose promotion materials would have higher uptake of COVID-19 vaccine booster dose.

## 2. Materials and Methods

### 2.1. Study Design

**Figure 1:**
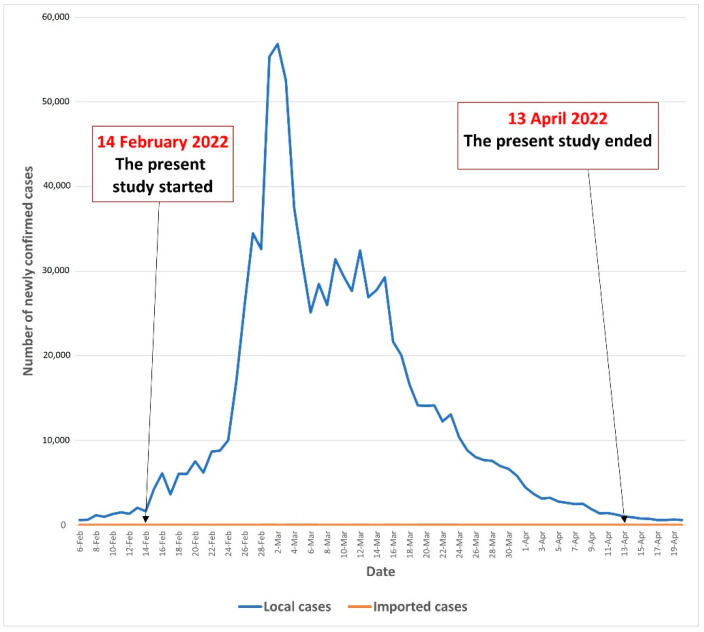
The COVID-19 situation in Hong Kong during the study period.

A random telephone survey was conducted among community-dwelling Chinese-speaking individuals aged ≥65 years in Hong Kong from 14 February to 13 April 2022. During the study period, Hong Kong was experiencing the fifth wave of COVID-19 outbreak caused by the Omicron BA.2. The number of daily confirmed COVID-19 cases was 1619 on 14 February 2022, and this peaked (*n* = 56,827) on 2 March 2022. By the end of this study (13 April 2022), daily confirmed cases dropped to 1043. We present the COVID-19 situation in Hong Kong during the study period in Figure 1.

### 2.2. Participants

The inclusion criteria were: (1) community-dwelling Chinese-speaking individuals aged ≥65 years, and (2) having a Hong Kong ID card. We excluded those who were not able to communicate effectively with the study interviewers.

### 2.3. Sample Size Planning

Our target sample size was 400. We assumed the uptake rate of the COVID-19 vaccine booster dose to be 50%. Assuming the uptake rate in the reference group (without a facilitating condition) to be 10–40%, the sample size could detect the smallest odds ratio of 1.76 between people with and without a facilitating condition (Power: 0.80, alpha value: 0.05; PASS 11.0, NCSS LLC). According to the Hong Kong census data in 2021, 18.2% of Hong Kong residents were 65 years or above [48]. Assuming the participation rate of valid households to be 55–60%, we needed to screen about 4000 households in order to recruit 400 eligible participants.

### 2.4. Data Collection

The research team input all household telephone numbers listed in the most up-to-date telephone directories (about 350,000) into an Excel file and randomly selected 4000 household telephone numbers by using the function of “select random cells”. Trained interviewers conducted the telephone calls during 6–10 p.m. on weekdays and 2–9 p.m. on Saturdays to avoid under-sampling of working individuals. If the initial call was not answered, four more follow-up calls were made during different time slots before we considered the household to be non-valid (one without an eligible participant). In case there was more than one person in the household who was aged ≥ 65 years, the interviewers invited the one whose last birthday was the closest to the interview date to complete the survey. This was to avoid data contamination and the introduction of extra confounding factors. After screening the eligibility, interviewers briefed prospective eligible participants about the study, and made guarantees of anonymity, the right to quit at any time, and that refusal would have no consequences. Since there was no face-to-face contact and the study was anonymous, we obtained verbal informed consent from the participants. The interviewers signed a form pledging that the participants had been fully informed about the study. The same procedures have been used in previous random telephone surveys targeting local older adults [45,49,50]. The telephone interview took about 20 min to complete. A total of 3900 households were called; 640 had an eligible older adult, 245 prospective participants refused to participate in the study, and 395 completed the telephone survey. The response rate was 62% (395/640). No incentives were provided to participants. Ethics approval was obtained from the Survey and Behavioral Research Ethics Committee of the Chinese University of Hong Kong (SBRE-19-187).

### 2.5. Measures

#### 2.5.1. Development of the Questionnaire

We performed in-depth interviews to understand older adults’ perspectives of a COVID-19 vaccine booster dose. Five community-dwelling Chinese-speaking individuals aged ≥ 65 years were recruited through purposive sampling (2 males and 3 females). Prior to the interview, fieldworkers explained the purposes and nature of the interviews. With verbal informed consent, interviews were conducted through telephone and audio recorded with informants’ consent. The interviews lasted for about one hour. We transcribed interviews and kept a code book to record special data and transform the data into categories to identify main themes. Three out of five informants intended to receive a COVID-19 vaccine booster dose in the future. We identified three themes related to facilitators: (1) concern that the protection conferred by the primary vaccine series would decline over time, (2) belief that a booster dose could reduce the risk of having Omicron BA.2 and the risk of death, and (3) suggestions from the government, doctor, and family members to receive a COVID-19 vaccine booster dose. Four other themes were related to barriers. They were: (1) not knowing which type of vaccine was better for them as a booster dose, (2) belief that older people would respond less well to a booster dose (less effective and more side effects), (3) concern about potential interactions between chronic diseases and a booster dose, and (4) concern about the duration of protection of a booster dose. Based on the qualitative findings, a panel of researchers in public health, behavioral health, and vaccination behaviors developed the questionnaire. The questionnaire was tested among 10 older adults to assess clarity and readability. All older adults participating in the pilot study believed that the questions were understandable and the length was acceptable. These older adults did not participate in the actual survey. The panel finalized the questionnaire based on the pilot testing results.

#### 2.5.2. Background Characteristics

Participants reported sociodemographic characteristics, the presence of chronic conditions, history of COVID-19, and history of seasonal influenza vaccination and pneumococcal vaccination.

#### 2.5.3. COVID-19 Vaccine Booster Dose Uptake

Participants reported the number of doses and types of COVID-19 vaccines received as primary vaccine series and the booster dose. Participants who had received a booster dose were asked for some details, such as the severity of side effects of the booster dose and whether such side effects were milder or more severe than the primary vaccine series.

#### 2.5.4. Perceptions Related to the COVID-19 Vaccine Booster Dose

We adapted the 3-item scale measuring positive attitudes toward a COVID-19 vaccine booster dose validated in the Chinese population [31]. One item of the original scale “China has sufficient supply of booster doses of COVID-19 vaccines” was replaced by “a booster dose is highly effective in preventing severe consequences of COVID-19”. We adapted the scale measuring negative attitudes toward the COVID-19 vaccine validated among older adults in Hong Kong [45]. We replaced the phrase “COVID-19 vaccine” with “COVID-19 vaccine booster dose” and added one new item to the original scale “the duration of protection offered by COVID-19 vaccine booster dose is shorter among people with older age” based on the feedback provided by older adults. The scales/items measuring perceived subjective norm, perceived behavioral control, and uncertainty related to COVID-19 vaccine booster dose were adapted from measurements validated in older Hong Kong adults [45]. We replaced the phrase “COVID-19 vaccine” with “COVID-19 vaccine booster dose”. The response categories for these measurements were 1 = disagree, 2 = neutral, and 3 = agree.

#### 2.5.5. Satisfaction with the COVID-19 Vaccine Booster Dose Health Promotional Materials Produced by the Government

We used the questions validated in Hong Kong older adults to assess satisfaction with COVID-19 vaccine booster dose health promotional materials produced by the Hong Kong government [45]. The questions were: (1) whether the information presented by these promotional materials was easy to understand, (2) whether these promotional materials could address their concerns related to a COVID-19 vaccine booster dose, and (3) whether these promotional materials could help them make the decision to receive the booster dose (responses categories: yes, no, and uncertain).

### 2.6. Statistical Analysis

The frequency distribution of all variables was presented. Mean and standard deviation (SD) of the items and scales representing perceptions related to the COVID-19 vaccine booster dose were also presented. Cronbach’s alphas were calculated by using reliability tests. We used principal component analysis with varimax rotation to perform exploratory factor analysis. The dependent variable was self-reported uptake of a COVID-19 vaccine booster dose. Univariate logistic regression models first assessed the significance of associations between background characteristics and the dependent variable. We then fitted a single logistic regression model involving all significant background characteristics and one independent variable of interest. A summary multiple logistic regression model was fitted considering all significant variables in univariate analysis. Crude odds ratios (OR), adjusted odds ratios (AOR), and their 95% confidence interval (CI) were obtained. In addition, correlations between perceptions and satisfaction with the health promotional materials were investigated. SPSS 26.0 (IBM Corp., Armonk, NY, USA) was used for data analysis, with *p* < 0.05 considered as statistically significant.

## 3. Results

### 3.1. Background Characteristics of the Participants

About half of the participants were 65–69 years (49.9%) and female (60.3%). The majority of them were married or cohabited with a partner (75.4%), did not receive tertiary education (89.9%), without a full-time or part-time work (85.8%), and had a monthly household income below HKD 20,000 (USD 2580) (73.9%). Over 60% of them had at least one chronic condition (60.8%). The most prevalent chronic condition was hypertension (47.6%), followed by diabetes mellitus (19.2%), chronic cardiovascular diseases (10.9%), and chronic lung diseases (10.9%). Among the participants, 10.6% reported a history of COVID-19, and 44.3% reported having an acquaintance with a history of COVID-19. At the survey time, 63.8% had received a seasonal influenza vaccination in their lifetime, and 42.5% received it both in the current and previous seasonal influenza seasons. In addition, 27.6% received pneumococcal vaccination in their lifetime (Table 1).

### 3.2. COVID-19 Vaccine Booster Dose Uptake

Among the participants, 31.6% (*n* = 125) received a COVID-19 vaccine booster dose. More participants chose Comirnaty (*n* = 65, 52%) rather than CoronaVac (*n* = 60, 48%) as their booster dose. Twelve participants mixed COVID-19 vaccine booster doses; eleven switched from CoronaVac to Comirnaty, and one switched from ZAD1222 to Comirnaty when receiving the booster dose. Over half of them reported no side effects of the COVID-19 vaccine booster dose, and 88.8% reported such side effects were about the same or even milder than those of their primary series (Table 2).

### 3.3. Perceptions Related to a COVID-19 Vaccine Booster Dose

The majority of the participants had positive attitudes toward the COVID-19 vaccine booster dose, such as the belief that receiving it could strengthen their protection against COVID-19 (74.4%), could effectively protect them from COVID-19 variants of concern (60.0%), and could prevent severe consequences of COVID-19 (82.5%). Relatively few participants were concerned that older age would lead to a lower level of protection (10.9%) and a higher level of side effects of the COVID-19 vaccine booster dose (14.2%). About 70% of them were sure about which type of the COVID-19 booster dose was suitable for them and which type they should choose. About 90% of the participants believed that governmental COVID-19 vaccine booster dose promotional materials were easy to understand (87.8%), 42.3% believed such materials could address their concerns related to the booster dose, and 48.6% perceived such materials as helpful for them when making the decision to receive the booster dose (Table 2).

### 3.4. Factors Associated with the COVID-19 Vaccine Booster Dose Uptake

In univariate analysis, higher education level and history of pneumococcal vaccination was associated with higher uptake of a COVID-19 vaccine booster dose (Table 3). After adjustment for significant background characteristics, more positive attitudes (AOR: 1.53, 95%CI: 1.24, 1.90), perceiving significant others would support them to receive the booster dose (AOR: 1.74, 95%CI: 1.35, 2.24), and less uncertainty when choosing a booster dose (AOR: 2.71, 95%CI: 1.94, 3.78) were associated with higher uptake of a COVID-19 vaccine booster dose. More negative attitudes toward the booster dose were negatively associated with the dependent variable (AOR: 0.83, 95%CI: 0.76, 0.90). Perceptions that governmental booster dose promotional materials could address their concern (AOR: 2.95, 95%CI: 1.89, 4.62) and help them to make a decision to receive the booster dose (AOR: 2.88, 95%CI: 1.83, 4.54) were also associated with higher uptake of the booster dose (Table 4).

Uncertainty Scale remained significantly associated with the dependent variable in the multiple logistic regression model (AOR: 2.25, 95%CI: 1.60, 3.17). However, education level, history of pneumococcal vaccination in the lifetime, positive or negative attitudes, perceived subjective norm, or satisfaction with the health promotional materials became statistically non-significant in the multiple logistic regression model (Table 5).

### 3.5. Correlations between Perceptions and Satisfaction with the Health Promotional Materials

Positive attitudes, perceived subjective norm, perceived behavioral control, and satisfaction with the health promotional materials were associated with less uncertainty, while negative attitudes were associated with higher uncertainty. Positive attitudes, perceived subjective norm, and perceived behavioral control were also associated with higher satisfaction with the health promotional materials, while negative attitudes were associated with lower satisfaction (Appendix A).

## 4. Discussion

This is one of the first studies examining facilitators and barriers of COVID-19 vaccine booster dose uptake among older adults in China. During the study period, 91.9% of participants received at least one dose of the COVID-19 vaccine, and 31.6% received a booster dose. Perceived threat of the ongoing outbreak of COVID-19 might motivate many older adults to complete their primary vaccine series and to receive the booster dose, as the proportion of taking up at least one dose of COVID-19 vaccine in this group was much higher than that observed between November 2021 and January 2022 (about 60%) [45]. The uptake rate of COVID-19 vaccine booster dose observed by our study was similar to the official record kept by the Hong Kong government during the same period (26% on 4 March 2022 and 39.6% on 13 April 2022 among people aged ≥ 60 years) [51,52]. However, the prevalence of COVID-19 vaccine booster dose uptake among our participants was lower than that of older adults in the United States (44% by December 2021) [32] and people aged 30–59 years in Hong Kong [51,52]. Older adults were the most vulnerable age groups during the COVID-19 pandemic [2]. Given the promising effectiveness of the booster dose in preventing severe consequences and deaths associated with COVID-19 [13,14,15], there is a strong need to increase COVID-19 vaccine booster dose coverage among older adults in Hong Kong.

Higher education level was positively associated with COVID-19 vaccine booster dose uptake among our participants. Such a finding was similar to those of previous studies, as higher education level was associated with a higher completion rate of primary COVID-19 vaccine series among older adults in Hong Kong [45] and higher intention to receive COVID-19 vaccine booster dose among Chinese factory workers [31]. Therefore, health communication messages promoting COVID-19 vaccine booster dose among older adults should be straightforward and easy to understand for people with low literacy levels. History of pneumococcal vaccination was also a facilitator to receive the COVID-19 vaccine booster dose in our sample. Previous studies suggested that history of pneumococcal vaccination was associated with higher uptake of primary COVID-19 vaccine series among older adults in Hong Kong, Italy, Canada, and Saudi Arabia [42,43,44,45]. It was possible that older adults with experiences of pneumococcal vaccination had stronger motivation to use vaccines to prevent infectious diseases.

This study provided some practical implications to increase coverage of the COVID-19 vaccine booster dose among older adults in Hong Kong. First, it is necessary to improve the existing COVID-19 vaccine booster dose promotional materials in Hong Kong. The information on these materials was friendly to older people, as about 90% of the participants found it easy to understand. However, about half of the participants thought such materials could not address their greatest concern related to the COVID-19 vaccine booster dose. A similar proportion of participants believed such materials were not helpful for them when it came to deciding to receive the booster dose. Since the satisfaction with the health promotional materials was a facilitator to receive the booster dose, it was important to improve the contents. Our studies revealed some concerns specific to older adults in Hong Kong. About 10–20% of the older adults in our study had concerns about interactions between old age, chronic conditions, and COVID-19 vaccine booster dose, such as whether older age and the presence of chronic conditions would result in poorer responses to COVID-19 vaccine booster dose (weaker and shorter duration of protection, and more severe side-effects). Local evidence showed that the protection against severe/fatal diseases and death conferred by the Comirnaty and CoronaVac was similar in older adults and their younger counterparts [15]. Moreover, the COVID-19 vaccine booster dose was safe for older adults [11,12]. Older people did not experience more side effects than their younger counterparts [11,12]. Furthermore, the presence of chronic conditions would not lead to more side effects of COVID-19 vaccination and there was no evidence showing that COVID-19 vaccination would affect chronic disease control. Health promotional materials should consider including this evidence to address older adults’ concerns. In addition, up-to-date evidence on the duration of protection conferred by the COVID-19 booster dose should be disseminated to older adults when it becomes available. Second, future health promotion should also strengthen older people’s positive attitudes toward the COVID-19 vaccine booster dose, as positive attitudes were associated with a higher uptake of the booster dose. Health communication messages should emphasize that older adults could benefit most from the booster dose compared to other age groups [14]. Third, involving family doctors and family members in health promotion might be another useful strategy, as their support was important for older adults to receive a COVID-19 vaccine booster dose. Fourth, the results also showed that over 30% of older adults were unsure which type of vaccine should be used as a booster dose. Less uncertainty about the choice was a facilitator to receive the booster dose. Results of the multiple logistic regression model further highlighted the importance of uncertainty. Efficacies and side-effects of different combinations of primary vaccine series and booster dose available in Hong Kong should be compared using tables or figures, which could help older adults to compare features across different options. Older adults who were more satisfied with governmental health promotional materials might be more knowledgeable about the COVID-19 vaccine booster dose. They might have attitudes that are more positive and fewer concerns about the COVID-19 booster dose, and hence less uncertainty about the choice of the booster dose. Future studies may test whether such a pathway exists.

Although this study was based on a random and population-based sample, it had several limitations. First, compared to census data [48], people who were 75 years or above were under-sampled by this study. However, the distribution of gender and age was similar to a recent random telephone survey among community-dwelling older adults between November 2021 and January 2022 [45]. Second, policies related to COVID-19 vaccination are being updated rapidly in Hong Kong in response to the pandemic. Our findings were most applicable to the situation during the COVID-19 outbreak. Third, this study did not include older residents of residential care homes. The findings might not be generalized to all older adults in Hong Kong. Fourth; selection bias existed due to non-response. Our response rate was comparable to random telephone surveys on vaccination behaviors among community-dwelling older adults [45,49,50]. Fifth, data were self-reported, and verification was not feasible. Social desirability bias and recall bias existed. Moreover, causality could not be established as this was a cross-sectional survey.

## 5. Conclusions

Community-dwelling older adults aged 65 years or above in Hong Kong reported a lower uptake of COVID-19 vaccine booster dose than their younger counterparts. Existing COVID-19 booster dose health promotional materials might not adequately address older adults’ main concerns. Such materials could be improved by addressing concerns about how older age and the presence of chronic conditions would affect responses to the COVID-19 vaccine booster dose. Strengthening positive attitudes, involving significant others of older adults, and reducing uncertainty about the choice of the booster dose might be useful strategies to increase the uptake of COVID-19 vaccine booster dose in this age group.

## Figures and Tables

**Table 1:** Background characteristics of the participants (*n* = 395).

Characteristics	N	%
**Sociodemographic characteristics**		
Age group, years		
65–69	197	49.9
70–74	132	33.4
75 or above	66	16.7
Gender		
Male	157	39.7
Female	238	60.3
Relationship status		
Currently single	97	24.6
Married or cohabited with a partner	298	75.4
Education level		
Primary or below	167	42.3
Secondary	188	47.6
Tertiary or above	40	10.1
Current employment status		
Unemployed/retired/housewife	339	85.8
Full-time/part-time	56	14.2
Monthly household income, HK$ (US$)		
<20,000 (2580)	292	73.9
≥20,000 (2580)	53	13.4
Refuse to disclose	50	12.7
Receiving Comprehensive Social Security Assistance (CSSA) ^1^		
No	364	92.2
Yes	31	7.8
Living alone		
No	328	83.0
Yes	67	17.0
**Presence of chronic conditions, yes**		
Hypertension	188	47.6
Chronic cardiovascular diseases	43	10.9
Chronic lung diseases	43	10.9
Chronic liver diseases	8	2.0
Chronic kidney diseases	2	0.5
Diabetes Mellitus	76	19.2
Any of above	240	60.8
**History of COVID-19 and COVID-19 vaccination**		
History of COVID-19		
No	353	89.4
Yes	42	10.6
Having an acquaintance with history of COVID-19		
No	220	55.7
Yes	175	44.3
Types of COVID-19 vaccine used in primary vaccination series		
Comirnaty (Fosun-BioNTech)	146	37.0
CoronaVac (Sinovac)	216	54.7
AZD1222 (Oxford AstraZeneca)	1	0.3
Had not received any COVID-19 vaccine	32	8.1
**History of other vaccination**		
History of seasonal influenza vaccination		
No	143	36.2
Only in the current seasonal influenza season (after October 2021)	11	2.8
Only in the previous seasonal influenza seasons (before October 2021)	73	18.5
Both in the current and previous seasonal influenza seasons	168	42.5
History of pneumococcal vaccination in lifetime		
No	286	72.4
Yes	109	27.6

^1^ CSSA: A governmental financial support scheme providing a safety net for those who cannot support themselves financially.

**Table 2:** Descriptive statistics of COVID-19 vaccine booster dose uptake and independent variables of interest (*n* = 395).

Variables	N	%
**Information related to COVID-19 vaccine booster dose**		
Uptake of COVID-19 vaccine booster dose		
No	270	68.4
Yes	125	31.6
Types of COVID-19 vaccine booster dose (among 125 participants who had received the booster dose)		
CoronaVac (primary series) + Comirnaty (booster dose)	11	8.8
AZD1222 (primary series) + Comirnaty (booster dose)	1	0.8
Comirnaty (primary series) + Comirnaty (booster dose)	53	42.4
CoronaVac (primary series) + CoronaVac (booster dose)	60	48.0
Side-effects of COVID-19 vaccine booster dose (among 125 participants who had received the booster dose)		
Not at all	64	51.2
Very mild	32	25.6
Mild	15	12.0
Moderate	7	5.6
Severe	6	4.8
Very severe	1	0.8
Comparing side-effects of the booster dose versus primary series		
Much milder	14	11.2
Somewhat milder	14	11.2
Same	73	58.4
Somewhat more severe	21	16.8
Much more severe	1	0.8
**Perceptions related COVID-19 vaccine booster dose**		
Positive attitudes toward COVID-19 vaccine booster dose, agree		
Receiving a booster dose can maintain your antibody level and strengthen the protection against COVID-19	294	74.4
A booster dose is highly effective in protecting your from COVID-19 variants of concern (e.g., Omicron, Delta)	237	60.0
A booster dose is highly effective in preventing severe consequences of COVID-19	326	82.5
Positive Attitude Scale ^1^, mean (SD)	8.0	1.3
Negative attitudes toward COVID-19 vaccine booster dose, agree		
The protection offered by COVID-19 vaccine booster dose is weaker among people with older age	43	10.9
The level of side-effects of COVID-19 vaccine booster dose is more severe among people with older age	56	14.2
The duration of protection offered by COVID-19 vaccine booster dose is shorter among people with older age	44	11.1
Presence of chronic diseases would decrease the protection of COVID-19 vaccine booster dose	95	24.1
COVID-19 vaccine booster dose would negatively influence the control of existing chronic conditions	69	17.5
Negative Attitude Scale ^2^, mean (SD)	8.4	2.8
Subjective norm related to COVID-19 vaccine booster dose, agree		
Hong Kong government support older adults to receive COVID-19 vaccine booster dose	382	96.7
Your family doctors would support you to receive COVID-19 vaccine booster dose	173	43.8
Your family members would support you to receive COVID-19 vaccine booster dose	257	65.1
Subjective Norm Scale ^3^, mean (SD)	8.0	1.0
Perceived behavioral control to take up COVID-19 vaccine booster dose, agree		
You are confident to receive COVID-19 vaccine booster dose	379	95.9
Item score, mean (SD)	2.9	0.3
Uncertainty about the choice of the booster dose, agree		
You are sure about which type of COVID-19 vaccine booster dose is suitable for you	268	67.8
You are sure about which type of COVID-19 vaccine booster dose you should choose	274	69.4
Uncertainty Scale ^4^, mean (SD)	5.3	1.1
**Satisfaction with COVID-19 vaccine booster dose health promotional materials (e.g., advertisement, poster and others) produced by the government**		
Whether the information are easy to understand		
No/uncertain	48	12.2
Yes	347	87.8
Whether the materials can address your concerns related to COVID-19 vaccine booster dose		
No/uncertain	228	57.7
Yes	167	42.3
Whether the materials are helpful for you to make decision to receive COVID-19 vaccine booster dose		
No/uncertain	203	51.4
Yes	192	48.6

^1^ Positive Attitude Scale: 3 items, Cronbach’s alpha: 0.69, one factor was identified by exploratory factor analysis, explaining 61.9% of total variance; ^2^ Negative Attitude Scale: 5 items, Cronbach’s alpha: 0.85, one factor was identified by exploratory factor analysis, explaining 62.9% of total variance; ^3^ Subjective Norm Scale: 3 items, Cronbach’s alpha: 0.63, one factor was identified by exploratory factor analysis, explaining 56.6% of total variance; ^4^ Uncertainty Scale: 2 items, Cronbach’s alpha: 0.94, one factor was identified by exploratory factor analysis, explaining 94% of total variance.

**Table 3:** Associations between background characteristics and COVID-19 vaccine booster dose uptake among older adults in Hong Kong (*n* = 395).

Characteristics	COVID-19 Vaccine Booster Dose Uptake (%)	OR (95%CI)	*p* Values
**Sociodemographic characteristics**			
Age group, years			
65–69	30.5	1.0	
70–74	38.6	1.44 (0.90, 2.29)	0.13
75 or above	21.2	0.62 (0.32, 1.19)	0.15
Gender			
Male	35.7	1.0	
Female	29.0	0.74 (0.48, 1.13)	0.16
Relationship status			
Currently single	30.9	1.0	
Married or cohabiting with a partner	31.9	1.05 (0.64, 1.71)	0.86
Education level			
Primary or below	25.1	1.0	
Secondary	35.6	1.65 (1.04, 2.61)	0.03
Tertiary or above	40.0	1.98 (0.96, 4.09)	0.06
Current employment status			
Unemployed/retired/housewife	30.4	1.0	
Full-time/part-time	39.3	1.48 (0.83, 2.66)	0.19
Monthly household income, HKD (USD)			
<20,000 (2580)	30.1	1.0	
≥20,000 (2580)	37.7	1.41 (0.76, 2.58)	0.27
Refuse to disclose	33.3	1.16 (0.61, 2.22)	0.66
Receiving Comprehensive Social Security Assistance (CSSA) ^1^			
No	33.0	1.0	
Yes	16.1	0.39 (0.15, 1.04)	0.06
Living alone			
No	32.6	1.0	
Yes	26.9	0.76 (0.42, 1.37)	0.36
Presence of any chronic conditions			
No	34.8	1.0	
Yes	29.6	0.79 (0.51, 1.21)	0.27
**History of COVID-19 and COVID-19 vaccination**			
History of COVID-19			
No	32.3	1.0	
Yes	26.2	0.74 (0.36, 1.53)	0.42
Having an acquaintance with history of COVID-19			
No	28.6	1.0	
Yes	35.4	1.37 (0.89, 2.09)	0.15
Types of COVID-19 vaccine used in primary vaccination series			
Comirnaty (Fosun-BioNTech)	36.3	1.0	
CoronaVac (Sinovac)	32.9	0.86 (0.55, 1.34)	0.50
AZD1222 (Oxford AstraZeneca)	100.0	N.A.	N.A.
Had not received any COVID-19 vaccine	0.0	N.A.	N.A.
**History of other vaccination**			
History of seasonal influenza vaccination in lifetime			
No	25.9	1.0	
Yes	34.9	1.54 (0.98, 2.42)	0.06
History of pneumococcal vaccination in lifetime			
No	27.3	1.0	
Yes	43.1	2.02 (1.28, 3.20)	0.003

OR—crude odds ratios; CI—confidence interval; N.A.—not applicable. ^1^ CSSA: A governmental financial support scheme providing a safety net for those who cannot support themselves financially.

**Table 4:** Factors associated with COVID-19 vaccine booster dose uptake among older adults in Hong Kong (*n* = 395).

Variables	OR (95%CI)	*p* Values	AOR (95%CI)	*p* Values
**Perceptions related COVID-19 vaccine booster dose**				
Positive Attitude Scale	1.58 (1.28, 1.95)	<0.001	1.53 (1.24, 1.90)	<0.001
Negative Attitude Scale	0.82 (0.75, 0.89)	<0.001	0.83 (0.76, 0.90)	<0.001
Subjective Norm Scale	1.80 (1.40, 2.31)	<0.001	1.74 (1.35, 2.24)	<0.001
Perceived behavioral control to take up COVID-19 vaccine booster dose	N.A.	N.A.	N.A.	N.A.
Uncertainty Scale	2.75 (1.96, 3.83)	<0.001	2.71 (1.94, 3.78)	<0.001
**Satisfaction with COVID-19 vaccine booster dose health promotional materials (e.g., advertisement, poster and others) produced by the government**				
Whether the information are easy to understand				
No/uncertain	1.0		1.0	
Yes	1.65 (0.81, 3.35)	0.17	1.74 (0.84, 3.64)	0.14
Whether the materials can address your concerns related to COVID-19 vaccine booster dose				
No/uncertain	1.0		1.0	
Yes	3.05 (1.97, 4.73)	<0.001	2.95 (1.89, 4.62)	<0.001
Whether the materials are helpful for you to make decision to receive COVID-19 vaccine booster dose				
No/uncertain	1.0		1.0	
Yes	3.07 (1.97, 4.80)	<0.001	2.88 (1.83, 4.54)	<0.001

OR—crude odds ratios; CI—confidence interval; AOR—adjusted odds ratios, odds ratios adjusted for significant background characteristics listed in Table 3; N.A.—not applicable.

**Table 5:** Summary model of factors associated with COVID-19 vaccine booster dose uptake among older adults in Hong Kong (*n* = 395).

Variables	AOR (95%CI)	*p* Values
Education level		
Primary or below	1.0	
Secondary	1.51 (0.90, 2.53)	0.12
Tertiary or above	1.49 (0.66, 3.36)	0.34
History of pneumococcal vaccination in lifetime		
No	1.0	
Yes	1.67 (0.98, 2.84)	0.06
Positive Attitude Scale	1.19 (0.93, 1.53)	0.16
Negative Attitude Scale	0.91 (0.82, 1.01)	0.08
Subjective Norm Scale	1.33 (0.98, 1.80)	0.06
Uncertainty Scale	2.25 (1.60, 3.17)	<0.001
Whether the materials can address your concerns related to COVID-19 vaccine booster dose		
No/uncertain	1.0	
Yes	1.52 (0.72, 3.23)	0.27
Whether the materials are helpful for you to make decision to receive COVID-19 vaccine booster dose		
No/uncertain	1.0	
Yes	1.24 (0.57, 2.69)	0.58

AOR—adjusted odds ratios, odds ratios obtained from multiple logistic regression model considering all significant variables in Table 3 and Table 4 as candidates.

## Data Availability

The data presented in this study are available from the corresponding author upon request. The data are not publicly available as they contain sensitive personal behaviors.

## Appendix A

**Table A1:** Correlations between perceptions and satisfaction of health promotional materials.

	Positive Attitude Scale	Negative Attitude Scale	Subjective Norm Scale	Perceived Behavioral Control	Decisional Conflict Scale	Whether the Information Are Easy to Understand	Whether the Materials Can Address Your Concerns	Whether the Materials Are Helpful for You to Make Decision
	r ^a^ (*p* Values)	r ^a^ (*p* Values)	r ^a^ (*p* Values)	r ^a^ (*p* Values)	r ^a^ (*p* Values)	r ^a^ (*p* Values)	r ^a^ (*p* Values)	r ^a^ (*p* Values)
Positive Attitude Scale	N.A.	−0.43 (*p* < 0.001)	0.38 (*p* < 0.001)	0.20 (*p* < 0.001)	0.16 (*p* = 0.001)	0.04 (*p* = 0.93)	0.18 (*p* < 0.001)	0.23 (*p* < 0.001)
Negative Attitude Scale	−0.43 (*p* < 0.001)	N.A.	−0.38 (*p* < 0.001)	−0.19 (*p* < 0.001)	−0.16 (*p* = 0.001)	0.01 (*p* = 0.40)	−0.15 (*p* = 0.003)	−0.14 (*p* = 0.004)
Subjective Norm Scale	0.38 (*p* < 0.001)	−0.38 (*p* < 0.001)	N.A.	0.31 (*p* < 0.001)	0.15 (*p* = 0.002)	0.08 (*p* = 0.10)	0.17 (*p* = 0.001)	0.22 (*p* < 0.001)
Perceived behavioral control	0.20 (*p* < 0.001)	−0.19 (*p* < 0.001)	0.31 (*p* < 0.001)	N.A.	0.17 (*p* = 0.001)	0.02 (*p* = 0.70)	0.12 (*p* = 0.02)	0.18 (*p* < 0.001)
Decisional Conflict Scale	0.16 (*p* = 0.001)	−0.16 (*p* = 0.001)	0.15 (*p* = 0.002)	0.17 (*p* = 0.001)	N.A.	0.17 (*p* = 0.001)	0.31 (*p* < 0.001)	0.28 (*p* < 0.001)
Whether the information are easy to understand	0.04 (*p* = 0.93)	0.01 (*p* = 0.40)	0.08 (*p* = 0.10)	0.02 (*p* = 0.70)	0.17 (*p* = 0.001)	N.A.	0.29 (*p* < 0.001)	0.25 (*p* < 0.001)
Whether the materials can address your concerns	0.18 (*p* < 0.001)	−0.15 (*p* = 0.003)	0.17 (*p* = 0.001)	0.12 (*p* = 0.02)	0.31 (*p* < 0.001)	0.29 (*p* < 0.001)	N.A.	0.78 (*p* < 0.001)
Whether the materials are helpful for you to make decision	0.23 (*p* < 0.001)	−0.14 (*p* = 0.004)	0.22 (*p* < 0.001)	0.18 (*p* < 0.001)	0.28 (*p* < 0.001)	0.25 (*p* < 0.001)	0.78 (*p* < 0.001)	N.A.

^a^ r: Pearson correlation coefficient. N.A.: not applicable
